# Cooked broiler meat quality affected by different *Mediterranean* medicinal plants in the diet

**DOI:** 10.5713/ab.21.0264

**Published:** 2021-08-25

**Authors:** Marwan A. AL-Hijazeen, Mustafa S. AL-Rawashdeh, Ghaid J. Al-Rabadi

**Affiliations:** 1 Department of Animal Production, Faculty of Agriculture, Mutah University, Karak 61710, Jordan

**Keywords:** Germander, Lipid Oxidation, Natural Antioxidant, Sensory Attributes, Oregano

## Abstract

**Objective:**

This study was conducted to investigate the effects of adding oregano (*Origanum syriacum* L.) and germander (*Teucrium polium* L.) to poultry diets individually and/or in combination: i) on cooked chicken meat quality and storage stability, ii) to compare this effect with those of the synthetic antioxidant butylated hydroxyanisole (BHA) and with the normal basic diet (Control: without supplements).

**Methods:**

Broilers (140 birds) were raised for 21 days and then equally divided into five different treatment groups of 28 birds each. The dietary treatments were as follows: i) control; ii) germander (GER, 1.5%); iii) oregano (ORE, 2.5%); iv) combination of GER and ORE (CM, 1.5%, and 2.5%); v) BHA (0.02%). Meat patties from the five treatments were prepared, cooked, and stored at 4°C prior to analysis. During storage, samples were measured for thiobarbituric acid-reactive substances (TBARS) and total carbonyl levels at 0, 4, and 7 days. In addition, cooked thigh meat was prepared separately to evaluate cooking loss and sensory attributes.

**Results:**

The CM dietary treatment showed the highest antioxidant effect, with decreasing TBARS values (breast and thigh meat) throughout the storage time (4 to 7 days). Furthermore, ORE showed a higher antioxidant effect, decreasing the rancidity development (TBARS values), compared to the GER during the storage period (days 0 to 7). The anti-carbonyl effect of the CM supplement was the highest among all treatments from day 0 to 7. Generally, the antioxidant effect of GER was lower compared to that of ORE and BHA alone. The CM treatment most significantly decreased off-odor and rancidity development, with the lowest oxidation odor scores.

**Conclusion:**

The results indicate that the combination of oregano and germander in the diet of boilers improves meat quality and prolongs shelf life.

## INTRODUCTION

Poultry meats are considered some of the best food protein sources, especially in the developed countries [1]. However, they are highly susceptible to auto-oxidation, which negatively affects their quality and shelf life [2,3]. The industry therefore uses types of chemical preservatives to maintain meat quality and safety [4,5], which are either directly added to the meat mixture [6–8] or indirectly by the animal feeding system [9–11]. Synthetic antioxidants, such as butylated hydroxyanisole (BHA) and ethoxyquin (generally recognizes as safe preservatives) are used in animal feedstuffs to prevent rancidity and to affect health, growth, and meat quality [12–14]. However, the addition of these supplements in broiler feed is considered unfavorable due to various side effects. For instance, the accumulation of these additives (residuals) in animal tissues (muscles) may have toxic or carcinogenic effects on human health [4]. Recent studies have therefore recommended to decrease the doses of these synthetic supplements and to replace them with natural alternatives [15]. For example, oregano essential oil has been used in broiler diets as a natural source of antioxidants; it is absorbed by muscle tissue and prevents fat oxidation [16]. Recently, the animal feed industry has become increasingly interested in natural alternatives (e.g., phytogenic feed additives, essential oils, probiotics, organic acids, prebiotics), which positively reflected in their product sales, feed quality, and overall acceptability by consumers [15]. In addition, changing poultry diets to be free of artificial additives is highly recommended to improve meat quality and to meet the requirements of the organic meat market [17]. Medicinal herbs are considered efficient alternatives to increase animal productivity [15,18]. For example, oregano, sage, and rosemary have shown positive effects on animal growth performance, health, and meat carcass quality [19–21]. These herbal supplements also affect broiler physiological feed absorption, digestibility, and use and have an impact on meat quality and the internal reducing capacity [15]. Their antioxidant, anticancer, and antibacterial properties provide the basis for these positive effects [22,23]. Furthermore, their effects on blood parameters (e.g., immunity and reduced capacity response) may enhance cellular reducing capacity (muscle cells) and prevent free radical formation [24]. For example, in a previous study, dietary oregano powder decreased the malondialdehyde (MDA) content and increased total antioxidant activity in the serum of broilers raised for 42 days [21]. In addition, muscle tissues also may absorb some of these compounds (phenols), similar to the vitamin E absorption mechanism, enhancing their internal reducing capacity [25]. The improvement in fresh meat reducing capacity is reflected in both cooked and processed meat products [3,4,26]. In previous studies, Jordanian wild oregano (*Origanum syriacum* L.) has been dried, and the oil has been extracted using different methods [27,28]. The final extract contains several polyphenolic compounds with antioxidant properties, such as *thymol* and *carvacrol* [27]. Another wild Jordanian herb which also contains several polyphenolic compounds is germander (*Teucrium polium* L.), which is used as a medicinal herb [23]. Analysis of Jordanian germander essential oil has revealed several compounds such as 8-cedren-13-ol (24.8%), B-caryophyllene (8.7%), sabinene (5.2%), and germacrene D (6.8%) [29]. In addition, flavonoids have a high antioxidant activity and 2.4-dinitrophenylhydrazine radical-scavenging activities, as found in Jordanian *Teucrium polium* (Ja’adeh) extracts; the highest antioxidant activity has been observed for luteolin-7-O-glucoside [22]. The phytochemical compounds isolated from *T. polium* have recently been classified and named as follows: i) volatile oils (germacrene D, up to 23%, β-caryophyllene (18%), β-pinene (18%), and α-pinene (12%), ii) compounds from *T. polium* aerial parts (isoprenoids, teuvincentins, and neo-clerodane diterpenoids), iii) compounds identified by gas chromatographic and spectroscopic techniques, iv) compounds in the aqueous phase (alcoholic extract), such as glucose and raffinose, v) major flavonoids, and vi) four sesquiterpenoid compounds identified by nuclear magnetic resonance spectroscopy [23].

*Origanum syriacum* and *Teucrium polium* are widely distributed in the south of Jordan [30,22,27] and contain high levels of phenolic compounds [27]. However, several factors may affect their antioxidant activities, such as storage conditions (e.g., temperature), cultivation season, time of cutting, and genetic variability [30,27]. There was no research studies conducted before to investigate this kind of plant combination on meat quality. In this context, the main objectives of this study were i). to investigate the effects of dietary oregano (*Origanum syriacum* L.) and germander (*Teucrium polium* L.) on broiler cooked meat quality and storage stability and ii) to compare their effects with those of synthetic antioxidant (BHA).

## MATERIALS AND METHODS

### Birds and diet formulation

The experimental design and all protocols involved were approved by the Animal Ethics Committee at the Department of Animal Production at Mutah University, Jordan (Ref: 123/14/120).

In total, 140 broilers were raised for 21 days (from 21 to 42 days old) according to general commercial husbandry practices. The broilers were randomly assigned to five dietary treatments (with each treatment containing seven replicates of four broilers each). The treatments were as follows: i) control (without supplement), ii) germander 1.5% (GER), iii) oregano 2.5% (ORE) iv) combination of GRE and ORE (CM) (1.5% and 2.5%, respectively), v) BHA (0.02%). Oregano (*Origanum syriacum* L. (locally known Za’tar) and germander *Teucrium polium* L. (locally known as Ja’ada) herbal plants were collected from the wild in the Al-Karak region of southern Jordan (150 km south of Amman). The plants were dried, ground into a fine powder, vacuum-packaged (impermeable oxygen bags), frozen, and stored until use. The BHA was dissolved and mixed with soybean oil (carrier material) to obtain a homogenous stock solution, according to the method of Al-Hijazeen [8]. During the experimental period, all birds were raised in floor cages and offered one of the five diets; water was provided *ad libitum* through drinkers. All diets were formulated to be isocaloric and isonitrogenous (Table 1).

### Meat preparation

At the end of the experiment period (at 6 weeks of age), all birds were fed a corn-soybean meal diet and slaughtered at Mutah University (Agriculture College/Department of Animal Production-farm facilities). All slaughtered birds were inspected and examined by veterinarians. The carcasses were cooled using ice water (1 h) and kept in the cold; subsequently, the muscles were deboned (breast and thigh), vacuum-packaged in oxygen-impermeable bags, and stored at −18°C until further analysis. Both breast and thigh meats were stored separately until use.

Raw meats (breast and thigh separately) were ground (twice) through an 8-mm and a 3-mm plate (Moulinex, Type DKA1, France), respectively, to prepare meat patties (50 g each) for all five treatments individually. The raw meat samples were packaged in oxygen-impermeable vacuum bags (Ehsan & Tahssin Baalbaki Co, Bayader Wadi Al-Seer, Amman, Jordan), and the meats were cooked in-bag in a 90°C water bath (Memmert, WNB 14; GMbH + Co. KH, Schwabach, Germany) until the internal temperature of the meat reached 75°C. After cooling, the cooked meat samples (50 g) were transferred to a new oxygen-permeable bag (polyethylene, 11×25 cm, Future for Plastic Industry, Al-Moumtaz bags, Co. L.T.D, Amman, Jordan) and stored at 4°C for 0, 4, and 7 days. Samples from each storage period were analyzed for thiobarbituric acid-reactive substances (TBARS) and total carbonyl amount. In addition, we measured cooking loss %, proximate composition, and ultimate pH using same preparation method. Furthermore, ground, meat patties of raw thigh samples were stored at 4°C up to 4 days before cooking to evaluate sensory attributes.

### pH of raw thigh meat

The ultimate pH values of the raw, ground meat samples were measured using a pH meter (PL-600, pH/mV/Temp Meter, Taipei, Taiwan) after homogenizing 1.0 g of the sample with 9 mL of deionized distilled water (DDW) [31].

### Cooking loss %

Chicken thigh meat samples (30 g) were weighed and packaged in oxygen-impermeable vacuum bags. The meat was cooked at a constant temperature in a pre-heated water bath (Memmert, WNB 14; GMbH + Co. KH, Germany) to the internal temperature of 80°C for 30 min to achieve maximum water loss [32]. After cooking, the meat samples were cooled in the water bath using cold water until reaching an internal temperature of 20°C and water-blotted or purged until dry. Cooking loss percentage was calculated as percent weight reduction of the cooked sample compared to the raw meat sample, using the following equation:


$$\text{Cooking loss }\%=\frac{\left(\text{Weight of raw meat}-\text{Weight of cooked meat}\right)}{\text{Weight of raw meat}}\times 100$$

### Proximate composition

All of treatments (fresh) meat batch were analyzed for their proximate compositions of fat, protein, water, and ash average percentage before cooking. Samples from each treatment (two sub-samples from each batch) (n = 4) were used according to standard methods [33].

### Thiobarbituric acid-reactive substances

All meat samples were assigned to the TBARS method [34] to evaluate lipid oxidation. The amounts of TBARS were expressed as mg of MDA per kg of meat.

### Protein oxidation (total carbonyl)

Protein oxidation was determined by estimating the total carbonyl value proposed by Lund et al [35], with minor modifications. The carbonyl content was calculated as nmol/mg protein, using the absorption coefficient of 22,000/M/cm as described by Levine et al [36].

### Sensorial analysis

A trained panel and the hedonic scale were used to evaluate the sensory characteristics of the cooked ground meat (thigh), as described by Al-Hijazeen et al [5]. The following parameters were analyzed: cooked meat color, spice odor (ORE and GER odor), oxidation odor, and overall acceptability. Five treatments were prepared as proposed in the oxidation analysis part to evaluate the effects of the different dietary supplements on chicken meat quality and storage stability.

The meat was refrigerated at 4°C (4 days) before cooking and for each evaluation session. Ten trained panelists (students and staff of Mutah University) participated in each session. Three 1-hour sessions were held using commercial and experimental products to train and develop descriptive terms for all attributes. All attributes were measured using a line scale without numbers (numerical value 9 units) from 0 to 9. Evaluation sessions for cooked meat samples were done at different days to avoid variability. The cooked samples (10 g each) were evaluated by the panelists for each treatment after cooling to 25°C. The panelists evaluated the content of one 20-mL glass vial of each treatment to estimate the odor of the cooked thigh meat sample. All sample vials were labeled with a random 3-digit number. After color evaluation, panelists were asked to open each vial in random order and evaluate the intensity of odor or the overall acceptability.

### Statistical analysis

Data were analyzed using the generalized linear model (Proc. GLM, SAS program; [37]). Mean values and standard error of the means were reported. The significance level was adopted at p<0.05, and we used Tukey’s test or Tukey’s multiple range to determine significant differences between the mean values.

## RESULTS AND DISCUSSION

### Cooking loss %, final pH, and proximate composition

The collected data showed no significant differences (p>0.05) in final pH mean values among control, GRE, ORE, and BHA treatments before storage at day 0 (Table 2). However, raw meat samples of the CM treatment showed the highest (p< 0.05) mean pH values (6.11) compared to the control, GRE, and BHA treatments. The higher final pH (24 hour) values favor meat color stability, water-holding capacity, shelf life, and various physio-chemical characteristics [38,39]. It is unclear why these values appeared in the birds fed these additives (GER and ORE), and future studies should investigate muscle metabolic changes. However, muscle glycolysis (glycolysis enzymes activity), free fatty acid, and glycogen content may cause this variation [40,41]. Several factors can affect broiler meat composition, such as diet ingredients, breed, and growing stage [42]. The supplements had no significant effect (p> 0.05) on the protein and ash average % of all raw meat samples. On the other hand, the lowest (p<0.05) fat percentage (5.78%) and the highest (p<0.05) water percentage (74.75%) were measured in the meat samples of birds fed CM supplement. However, there were no significant differences (p>0.05) among the samples in terms of cooking loss %.

So far, only few studies have evaluated the effects of the addition of oregano [43,24,21] and germander on meat composition and quality. For example, Ri et al [21] investigated the effects of oregano powder (150 mg/kg) on the growth performance, antioxidant status, and meat quality of broiler chickens. Similar to our study, the authors found no effects on carcass yield, cooking and dripping losses, shear force, pH value, and meat color.

### Lipid oxidation

Generally, lipid autoxidation depends on fresh meat status, free radical formation, and initiation reactions [3]. However, higher TBARS values in the fresh meat will cause further deterioration of the cooked meat [6,7]. In the current study, all supplements decreased the initial (day 0) TBARS values compared to the control (Table 3). In previous studies, the antioxidant properties of these dietary supplements enhanced muscle reducing capacity and, consequently, autoxidation reactions in the cooked meat [43,3,24]. In addition, the initial TBARS values may also be affected by meat composition and metabolism. In one study, the addition of vitamin E at the same level as that of oregano oil (100 mg/kg) resulted in a more pronounced antioxidant effect on cooked chicken meat (ground meat patties) compared to the other treatment including BHA (0.02%) [16]. In addition, the absorption of plant phenolic compounds occurs similarly as vitamin E mechanism reported [24]. However, CM had the highest (p<0.05) effect compared to the other treatments for both breast and thigh meats. There were no significant differences (p>0.05) between both ORE and GER samples at day 0 of the storage period. Furthermore, ORE showed a higher effect in decreasing the TBARS values compared to GER. The BHA effect was similar for both meat types (breast and thigh) at day 0 compared to the control samples. All dietary supplements significantly (p<0.05) delayed malonylaldehyde formation after 4 days compared to the control treatment, most likely because of the initial amount of free radicals in all samples. This is in agreement with the findings of previous studies regarding the increase in muscle reducing capacity of fresh meat with the addition of dietary vitamin E [16]. According to a previous study, dietary oregano powder enhances superoxide dismutase and glutathione peroxidase enzyme activities and causes TBARS values of duck meat [24]. The authors also reported that some phenolic compounds are absorbed by the muscle tissues of ducks, supporting the antioxidant defense system. However, this variation among treatments maximized and became more significant with the increase in the TBARS value. The antioxidant activity of these plant supplements generally depends on their constituents (phenols and others) and concentrations [27,28]. In addition, several other benefits could be achieved when using these plants as dietary supplements. For instance, essential oils of these plants have successfully been used as dietary antibiotics, without residual effects [44]. Generally, the CM treatment showed the highest decrease in TBARS values during different storage periods compared to the other supplements. Finally, there were no significant differences (p>0.05) between ORE and CM treatments in terms of TBARS values after 7 days of storage, irrespective of the meat type. In addition, the effect of GER alone was lower compared to that of the other supplements during the storage period. No synergistic effect was found by adding a combination of oregano and germander, indicating that ORE was the most effective supplement reducing TBARS values. Finally, the antioxidant activity of these supplements was higher when they were used in combination instead of individually.

### Protein oxidation

The total initial carbonyl values were slightly higher in the control meat samples. The values were similar compared to those found in previous studies, where up to 5 nmol/mg protein of the cooked meat products were measured [45,46]. In addition, CM and ORE samples showed the lowest values compared to the control and other treatments (GER and BHA) at day 0 of storage for both meat types (Table 4). This was similar as the lipid oxidation proceeded and was affected by these supplements. In addition, this effect was also linked with lipid oxidation (primary and secondary products) and interaction with protein oxidation [47]; these protein-lipid interactions significantly affect protein functional properties and, consequently, the quality of the final product [46,48]. In addition, the reducing capacity obtained via these supplements in the fresh meat decreased the total carbonyl values, which is linked with muscle absorption of plant phenolic compounds [49,43,24]. The effect of dietary germander was lower compared to those of ORE and BHA alone. However, this effect increased when germander was combined with the oregano supplement. The antioxidant effect of CM was highest during different storage periods.

### Sensory evaluation

Only few studies have evaluated the effects of dietary oregano (*Origanum syriacum* L.) [9,49,43] combined with GER (*Teucrium polium* L.) supplements on poultry meat quality and sensory characteristics. A decrease in off-odor volatiles, rancidity, and positive overall acceptability, as well as low TBARS and total carbonyl values in the meat have been reported by Al-Hijazeen et al [50] when studying the effect of adding oregano and tannic acid combinations on the quality and sensory characteristics of cooked chicken meat. The only feed supplement which had a significant (p<0.05) effect on the cooked meat color was GER. However, there were no significant differences (p>0.05) among the other treatments. The addition of these supplements to broiler diets had an impact on the spice-odor attributes of cooked thigh meat samples. In addition, the CM treatment showed the highest (p<0.05) score values regarding spice odor (Table 5). No significant differences were found between GER and ORE. According to the panelists, combination treatment most significantly decreased the off-odor and rancidity development, with the lowest oxidation-odor scores. This is in agreement with current reported TBARS and total carbonyl results, in particular for the CM treatment. Furthermore, all supplements had a significant (p<0.05) effect on oxidation odor. Finally, ground cooked meat samples from the ORE, BHA, and CM treatments showed the highest overall acceptability compared to the other treatments. However, dietary germander negatively impacted odor and taste (bitter taste; data not shown) as well as cooked meat color.

## CONCLUSION

Based on current findings, dietary GER and ORE positively affected meat storage stability. Adding these supplements impacted overall muscle fat deposition, metabolism, and ultimate pH values, with no significant effect on cooking loss %. Current study showed positive effect on meat shelf life by decreasing the TBARS and total carbonyl values. In addition, there were positive effects on most sensorial attributes of the cooked meat. Generally, the antioxidant effects of ORE were better than those of GRE. However, these effects could be maximized by combining ORE and GRE in broiler diets. Our results lead us to infer that the addition of CM to broiler diets is a promising approach in the poultry industry to improve meat stability.

## Figures and Tables

**Table 1:** Ingredients and chemical compositions (%) of the treatment diets

Item	Treatment^1)^				

	Control	GER	ORE	CM	BHA
Ingredients (%)					
Corn	49.97	50.85	51.2	51.72	49.97
Soybean meal	30.56	33.25	33.9	34.87	30.53
DL-methionine	0.05	0.07	0.07	0.07	0.05
Limestone	1.54	1.69	1.69	1.69	1.53
Soybean oil	6.00	6.00	6.00	6.00	6.00
Salts	0.27	0.33	0.34	0.34	0.27
Minerals and Vit premix^2)^	0.10	0.10	0.10	0.10	0.10
Antifungi^3)^	0.00	0.00	0.00	0.00	0.00
Monocalcium phosphate	0.66	0.84	0.84	0.86	0.66
Bronocon concentrate^4)^	2.06	0.00	0.00	0.00	2.12
Germander	0.00	1.50	0.00	1.50	0.00
Oregano	0.00	0.00	2.50	2.50	0.00
BHA	0.00	0.00	0.00	0.00	0.02
Wheat bran	8.49	5.08	3.07	0.07	8.46
Threonine	0.30	0.28	0.28	0.28	0.30
Chemical composition (%)^5)^					
DM (%)	90.00	90.20	90.30	90.40	90.00
ME (Mcal/kg)	3050	3050	3050	3050	3050
Crude protein	20.00	20.00	20.00	20.00	20.00
Crude fiber	4.27	4.00	3.84	3.59	4.26
Methionine	0.38	0.38	0.38	0.38	0.38
Lysine	1.054	1.05	1.064	1.073	1.054
Calcium	0.90	0.90	0.90	0.90	0.90
Non phytate phosphorus	0.35	0.35	0.35	0.35	0.35

1) Treatments: Control, without supplements; GER, germander; ORE, oregano; CM, combination; BHA, butylated hydroxyanisole.

2) Vitamin premix provided per kilogram of premix: vitamin A,700,000 IU; vitamin D_3_, 150,000 IU; vitamin E, 75mg; vitamin B_1_, 100 mg; vitamin K, 175 mg; vitamin B_5_, 600 mg; manganese oxide, 4,000 mg, ferrous sulphate, 9,000 mg, zinc oxide, 6,000 mg, magnesium oxide, 2,500 mg, potassium iodide, 70 mg, sodium selenite, 125 mg, copper sulphate, 100 mg, cobalt sulphate, 50 mg, dicalcium phosphate, 7,000 m, sodium chloride, 10,000 mg.

3) Mold inhibitor for animal feed (Kemin Industries, USA).

4) Brocon Concentrate (Wafa, B V, Alblasserdam, Holland) provide (%, as on feed basis): metabolizable energy, 2,200 kcal/kg; crude protein, 35%; crude fiber, 4.8; non-phytate phosphorus, 2.2%; methionine, 1.6%; lysine, 2.4%; cysteine, 0.3%.

5) Formulated diets were calculated on the basis of analyzed values of feed ingredients (feed composition tables) from poultry NRC (1994).

**Table 2:** Cooking loss %, pA^1)^, and U- pH^2)^ values of ground chicken thigh meat of all treatment samples

TRT^1)^	Cooking loss %	Proximate analysis				U-pH values

		Fat %	Protein %	Water %	Ash%	
Control	0.181	7.23^a^	18.52	73.35^c^	0.90	5.77^b^
GRE	0.175	6.85^b^	18.50	73.73^c^	0.92	5.85^b^
ORE	0.169	6.14^c^	18.68	74.28^b^	0.90	5.88^ab^
CM	0.165	5.78^d^	18.54	74.75^a^	0.93	6.11^a^
BHA	0.177	6.94^b^	18.44	73.70^c^	0.92	5.79^b^
SEM	0.013	0.039	0.083	0.095	0.017	0.057

SEM, standard error of the mean.

1) pA, proximate analysis of fresh meat before cooking.

2) U-pH, ultimate pH of raw meat.

3) Treatments: Control, without supplements; GER, germander; ORE, oregano; CM, combination; BHA, butylated hydroxyanisole. n = 4.

a–d Values with different letters within a column are significantly different (p<0.05).

**Table 3:** TBARS values of cooked ground meat at different storage periods at 4°C

Storage time	Treatment^1)^					SEM

	Control	GER	ORE	CM	BHA	
Breast	--------------------------------------------------------------- TBARS (mg/kg) meat----------------------------------------------------------					
Day 0	0.23^a,z^	0.21^ab,z^	0.11^bc,z^	0.18^c,z^	0.21^abc,z^	0.006
Day 4	1.69^a,y^	1.15^b,y^	0.89^cd,y^	0.73^d,y^	0.99^bc,y^	0.041
Day 7	2.14^a,x^	1.78^b,x^	1.32^cd,x^	1.15^d,x^	1.46^c,x^	0.069
SEM	0.041	0.041	0.035	0.061	0.05	
Thigh						
Day 0	1.20^a,z^	1.02^ab,z^	0.9^bc,z^	0.57^c,z^	0.97^abc,z^	0.061
Day 4	3.40^a,y^	2.48^b,y^	1.55^d,y^	1.3^d,y^	2.15^c,y^	0.061
Day 7	6.87^a,x^	3.90^b,x^	2.42^d,x^	2.14^d,x^	3.15^c,x^	0.126
SEM	0.080	0.109	0.091	0.077	0.079	

TBARS, thiobarbituric acid-reactive substances (mg malonaldehyde/kg meat); SEM, standard error of the mean.

1) Treatments: Control, without supplements; GER, germander; ORE, oregano; CM, combination; BHA, butylated hydroxyanisole.

a–d Values with different letters within a row are significantly different (p<0.05). n = 4.

x–z Values with different letters within a column are significantly different (p<0.05).

**Table 4:** Total carbonyl values of cooked ground meat using different dietary additives

Storage time	Treatment^1)^					SEM

	Control	GER	ORE	CM	BHA	
	-------------------------------------------------------- Carbonyl (nmol/mg of protein) --------------------------------------------------					
Breast						
Day 0	0.985^a,z^	0.861^ab,y^	0.813^bc,y^	0.691^c,y^	0.811^bc,z^	0.0385
Day 4	1.587^a,y^	1.208^b,xy^	1.005^bc,y^	0.937^c,xy^	1.08^bc,y^	0.0613
Day 7	2.229^a,x^	1.704^ab,x^	1.302^b,x^	1.136^b,x^	1.327^b,x^	0.141
SEM	0.062	0.156	0.066	0.085	0.042	-
Thigh						
Day 0	1.55^a,z^	1.46^ab,y^	1.23^cd,y^	1.16^d,z^	1.38^bc,z^	0.038
Day 4	3.17^a,y^	2.92^ab,x^	2.27^c,x^	1.97^c,y^	2.69^b,y^	0.081
Day 7	3.89^a,x^	3.23^b,x^	2.6^d,x^	2.37^e,x^	2.93^c,x^	0.053
SEM	0.030	0.079	0.086	0.046	0.035	-

SEM, standard error of the mean.

1) Treatments: Control, without supplements; GER, germander; ORE, oregano; CM, combination; BHA, butylated hydroxyanisole.

a–c Values with different letters within a row are significantly different (p<0.05). n = 4.

x–z Values with different letters within a column are significantly different (p<0.05).

**Table 5:** Sensory attributes: mean values of cooked ground (thigh) meat patties

TRT^2)^	Sensory attribute^1)^			

	Cooked Color	Spice Odor	Oxidation Odor	Overall Acceptability
Control	5.49^a^	0.73^c^	7.07^a^	4.75^c^
GER	3.79^b^	5.08^b^	5.05^b^	5.58^bc^
ORE	6.95^a^	5.44^b^	3.86^bc^	7.33^a^
CM	5.70^a^	7.09^a^	3.17^c^	6.85^ab^
BHA	6.87^a^	0.52^c^	4.26^bc^	6.12^ab^
SEM	0.384	0.239	0.362	0.334

SEM, standard error of the means.

1) Sensory attributes: samples were evaluated on day 3.

2) Control, without supplements; GER, germander; ORE, oregano; CM, combination; BHA, butylated hydroxyanisole. n = 10.

a–c Means within the same column with different superscripts are different (p<0.05).
